# Supplementary material for: Global hypo-methylation in a proportion of glioblastoma enriched for an astrocytic signature is associated with increased invasion and altered immune landscape
Source: eLife. 2022 Nov 22;11:e77335. doi: 10.7554/eLife.77335 (PMC9681209; doi:10.7554/eLife.77335)
Supplement: Figure 2—source data 1. [file elife-77335-fig2-data1.zip › Figure_2_source_data_1/Figure_2I_J/homerResults/motif45.similar.html]

motif45

## Information for motif45

T
A
C
G
A
C
T
G
T
A
C
G
T
C
A
G
A
T
C
G
T
G
A
C
A
T
C
G
A
C
G
T
A
T
C
G
A
T
C
G
T
A
G
C
T
G
A
C
T
A
G
C
C
T
A
G
T
A
C
G
C
A
T
G
T
A
C
G
T
A
G
C
T
G
A
C
T
A
C
G
T
C
A
G
T
C
A
G
T
A
C
G
A
T
G
C
T
A
G
C
T
G
A
C
T
A
C
G
A
T
C
G
A
T
C
G
T
G
C
A
T
A
C
G
A
G
T
C
T
A
C
G
G
T
A
C
A
T
G
C
T
G
C
A
A
T
G
C
A
T
G
C
A
C
T
G
A
T
C
G
T
A
C
G
T
G
A
C
A
T
G
C
A
G
T
C
A
T
G
C
A
G
T
C
A
T
C
G
T
A
C
G
T
A
G
C
T
A
G
C
  
Reverse Opposite:  

A
T
C
G
A
T
C
G
A
T
G
C
T
A
G
C
T
C
A
G
T
A
C
G
T
C
A
G
T
A
C
G
A
C
T
G
A
T
G
C
T
A
G
C
T
G
A
C
T
A
C
G
T
A
C
G
A
C
G
T
T
A
C
G
C
A
T
G
A
T
G
C
T
C
A
G
A
T
G
C
A
C
G
T
A
T
G
C
T
A
G
C
A
T
G
C
A
C
T
G
A
T
C
G
T
A
C
G
A
T
G
C
A
G
T
C
A
G
T
C
A
T
G
C
A
C
T
G
A
T
C
G
A
T
G
C
G
T
A
C
A
T
G
C
G
A
T
C
A
T
C
G
A
C
T
G
A
T
C
G
T
A
G
C
T
A
G
C
T
G
C
A
T
A
G
C
A
C
T
G
T
A
G
C
A
G
T
C
A
T
G
C
T
G
A
C
A
T
G
C
  

|  |  |
| --- | --- |
| p-value: | 1e-21 |
| log p-value: | -4.929e+01 |
| Information Content per bp: | 1.464 |
| Number of Target Sequences with motif | 30.0 |
| Percentage of Target Sequences with motif | 4.13% |
| Number of Background Sequences with motif | 1.7 |
| Percentage of Background Sequences with motif | 0.61% |
| Average Position of motif in Targets | 110.3 +/- 51.1bp |
| Average Position of motif in Background | 90.8 +/- 29.2bp |
| Strand Bias (log2 ratio + to - strand density) | 0.7 |
| Multiplicity (# of sites on avg that occur together) | 1.13 |
| Motif File: | file (matrix) reverse opposite |

### Similar de novo motifs found

|  |  |  |  |  |  |  |  |
| --- | --- | --- | --- | --- | --- | --- | --- |
| Rank | Match Score | Redundant Motif | P-value | log P-value | % of Targets | % of Background | Motif file |
| 1 | 0.601 | A T C G T A C G A T G C T A G C A T G C T G A C A T C G A T C G T C A G T A C G T A C G T A C G T A G C T C G A T A C G A T C G A T C G A T G C A T G C A C T G C T A G T A C G C T A G T A C G T G A C T C A G T A C G C T A G C T A G A T C G | 1e-15 | -34.587662 | 4.41% | 0.79% | motif file (matrix) |
